# Polyplexes of Functional PAMAM Dendrimer/Apoptin Gene Induce Apoptosis of Human Primary Glioma Cells In Vitro

**DOI:** 10.3390/polym11020296

**Published:** 2019-02-10

**Authors:** Yoonhee Bae, Le Thi Thuy, Young Hwa Lee, Kyung Soo Ko, Jin Han, Joon Sig Choi

**Affiliations:** 1 Department of Physiology, College of Medicine, Cardiovascular and Metabolic Disease Center, Inje University, Busan 47392, Korea; yoonhee-1@hanmail.net; 2 Department of Biochemistry, College of Natural Sciences, Chungnam National University, Daejeon 305-764, Korea; ltthuy1990@gmail.com (L.T.T.); youngwonho@naver.com (Y.H.L.); 3 Department of Internal Medicine, Sanggye Paik Hospital, Cardiovascular and Metabolic Disease Center, Inje University, Seoul 100-032, Korea; kskomd@hanmail.net

**Keywords:** PAMAM-FHR, apoptin, apoptosis, cancer therapy

## Abstract

Highly efficient and safe gene delivery has become an important aspect of neuronal gene therapy. We evaluated the ability of polyamidoamine (PAMAM) dendrimer grafted with phenylalanine, histidine, and arginine (PAMAM-FHR), a nonviral gene delivery vector, to deliver a therapeutic, tumor cell-specific killer gene, apoptin, into the human primary glioma cell line GBL-14 and human dermal fibroblasts. We performed a transfection assay using plasmids of luciferase and enhanced green fluorescent protein (EGFP) and assessed cell viability. Both cell lines were treated with complexes of PAMAM-FHR and apoptin after which their intracellular uptake and localization were examined by fluorescence-activated cell sorting (FACS)analysis and confocal laser scanning microscopy. Confocal microscopy showed that the PAMAM-FHR escaped from the endo-lysosome into the cytosol. Cell cycle phase distribution analysis, annexin V staining, and a tetramethylrhodamine ethyl ester (TMRE) assay established that apoptin triggered apoptosis in the GBL-14 cell line but not in normal fibroblasts. These results indicated that the PAMAM-FHR/apoptin complex is an effective gene vehicle for cancer therapy in vitro.

## 1. Introduction

Glioblastoma multiform (GBM) tumors are the most lethal malignant brain tumors in adults. GBM is characterized by cellular proliferation, tumorigenicity, and genomic instability [1,2]. Treatment options include surgery, cytotoxic therapy, radiotherapy, and temozolomide (TMZ) administration and their choice depends on the grade of malignancy. However, resistance to therapeutic agents and tumor reoccurrence at the primary site after the completion of therapy are still major issues [3,4]. Therefore, new strategies are needed for brain tumor diagnosis and treatment. One such approach is targeted gene therapy, which replaces a deficient gene by transferring genetic material into the target cells or tissues [5,6]. Most gene therapy approaches are based on the use of viral vector systems. Although viral vectors result in high gene expression, they have serious drawbacks including high toxicity, immunogenicity, and mutagenicity [7,8]. Therefore, nonviral vectors have attracted attention because of their higher biocompatibility, easier manufacturing, and low immunogenicity compared to a viral vector. Among nonviral vectors, cationic hyperbranched glycoconjugated polymers are important for gene therapy as multifunctional gene vectors due to low cytotoxicity and high transfection efficiency and gene therapy. Nonviral vectors for gene delivery include liposomes, cationic polymers, polypeptides, cationic oligomer, and inorganic nanoparticles [9,10]. In particular, biopolymer-based gene vectors are the best candidates for therapeutic applications due to their negligible toxicity, low immune reaction, and biocompatibility [11,12]. 

Polyamidoamine (PAMAM) dendrimers are synthetic cationic polymers with a flexible structure and are widely used as nonviral gene and drug delivery carriers because they result in high gene expression and low immunogenicity compared to other cationic polymers [13,14]. The primary and tertiary internal amine groups in PAMAM are protonated and form complexes with negatively charged deoxyribonucleic acid (DNA) via electrostatic interactions. These complexes effectively induce intracellular internalization by endocytosis [15,16]. As shown in one of our previous reports, histidine (H) and arginine (R)-conjugated PAMAM, PAMAM-H-R, demonstrated a higher buffer capacity compared to PAMAM. The effect overcame the acidic environment of the lysosome resulting in enhanced endosomal release and improved luciferase activity with low cytotoxicity [17]. Recently, we have shown that the incorporation of the hydrophobic amino-acid phenylalanine (F) into PAMAM-H-R, resulting in PAMAM-FHR, exhibits increased binding to the lipid bilayer of the membranes. When the PMAMA-FHR/DNA complexes enter the cells, the polymer peptide bonds are hydrolyzed by peptidases, facilitating DNA release within the cell by virtue of the positive charge density. The subsequent membrane disruption results in rapid endosomal escape into the cytosol and increased effectiveness of the gene delivery carrier [18,19,20]. Moreover, previous reports showed that the influenza virus-inspired polymer carrier, A-C3, undergoes endosomal escape allowing for the release of siRNA via self-catalyzed polymer degradation without external release stimuli inducing pH, light, enzyme degradation, and redox potential [21,22]. In this study, we aimed to develop a cancer therapeutic strategy using a cancer-selective killer gene, apoptin, and a nonviral delivery system for the treatment of a patient-derived human glioma GBL-14 cell line. 

Apoptin, encoded by the chicken anemia virus (CAV), VP3 gene, consists of 121 amino acids comprising proline, serine, and threonine and contains a bipartite nuclear localization sequence (NLS) and a nuclear export sequence (NES). Apoptin induces apoptosis specifically in tumorigenic cell lines but not in normal human cell lines [23,24]. The cancer-selectivity of apoptin is due to its differential intracellular distribution, as it predominantly accumulates in the nucleus of transformed and cancer cells, whereas it is cytosolic in normal cells. However, the mechanisms underlying apoptin-induced apoptosis are still unclear. According to one hypothesis, Thr 108 phosphorylation by an apoptin-specific kinase, mitogenic cyclin-dependent kinase (CDK2), induces apoptosis by cancer-specific nuclear translocation of apoptin in malignant cells. Therefore, apoptin may potentially be employed as a selective anticancer agent and various trials are ongoing to test this application [25,26]. 

The goal of this study was to test the suitability of PAMAM-FHR as a highly efficient apoptin gene delivery vector for therapeutic application. Thus, we evaluated cytotoxicity and gene transfection of PAMAM-FHR/DNA complexes. Moreover, we employed FACS analysis and confocal laser scanning microscopy to investigate the intracellular transport and localization of the complexes. The effect of PAMAM-FHR-mediated transfer of the apoptin gene into GBL-14 and human dermal fibroblasts were evaluated by annexin V staining, cell cycle analysis, and JC-1 assays. The results demonstrated that the PAMAM-FHR/pJDK-apoptin complexes showed reduced cytotoxicity, improved gene transfection efficiency, and rapid endolysosomal escape and caused cell death by inducing mitochondrial membrane permeability in human primary glioma cells but not in normal fibroblasts.

## 2. Materials and Methods

### 2.1. Materials

PAMAM, trifluoroacetic acid (TFA), *N*,*N*-diisopropylethylamine (DIPEA), dimethyl sulfoxide (DMSO), *N*,*N*-dimethylformamide (DMF), and PEI25KD were obtained from Sigma-Aldrich (Seoul, Korea).

### 2.2. Synthesis of PAMAM-FHR

PAMAM-FHR was synthesized as reported previously [18]. The overall synthesis scheme is shown in Figure S1. The ^1^H NMR of PAMAM-FHR is displayed in Figure S2. 

### 2.3. Plasmids and Cell Culture

Plasmids used for the experiments were kindly supported by J.S. Park [27]. GBL-14 and U373-MG cell lines, as well as human dermal fibroblasts, were cultured in DMEM plus 10% FBS and 100 unit/mL antibiotic-antimycotic agents (Invitrogen, Seoul, Korea).

### 2.4. Gel Retardation Assay

Agarose gel electrophoresis was preformed using A 10 mM HEPES buffer. DNA (0.5 µg/μL) was complexed with PAMAM derivatives (1 mg/mL) and pJDK or pJDK-apoptin at various weight ratios (0 to 8) in a total volume of 10 µL of water before being incubated for 30 min at 25 °C. The complexes were subjected to electrophoresis on 0.7% agarose gel with 0.5 μg/mL ethidium bromide (EB) at 100 V for 30 min and visualized by UV light (Bio-Rad, Seoul, Korea). 

### 2.5. PicoGreen Assay

The complex was prepared as per the agarose gel retardation assay. PicoGreen reagent was diluted in Tris-EDTA buffer and each sample was incubated for 30 min at 25 °C and each complex was mixed with PicoGreen reagent and further incubated for 2 min at 25 °C. Fluorescence intensity was performed using a fluorometer (Thermo Scientific, MA, USA). 

### 2.6. Cytotoxicity Assay

GBL-14 (1.3 × 10^4^), U373-MG (1.3 × 10^4^), and dermal fibroblasts (1.3 × 10^4^) were cultured in a 96-well tissue culture plate. The cytotoxicity activity was assessed by the EZ-Cytox assay kit (Daeil Lab Service, Seoul, Korea) according to the manufacture’s protocol.

Lactate dehydrogenase (LDH) toxicity assays were performed using a Lactate Dehydrogenase Assay Kit (Daeil Lab Service, Seoul). GBL-14 (1.3 × 10^4^), U373-MG (1.3 × 10^4^), and dermal fibroblasts (1.3 × 10^4^) were seeded in 96-well tissue culture plates. The amount of LDH release by cells was determined according to the manufacturer’s instructions and the absorbance was measured using an ELISA plate reader (Molecular Devices, USA).

### 2.7. Cellular Uptake Imaging

GBL-14 (5 × 10^3^) and dermal fibroblasts (5 × 10^3^) were cultured in 15 μ-slide 8-well plates (Ibidi, Seoul, Korea) and incubated with (Alexa Fluor 546-labeled pJDK or pJDK-apoptin (0.5 μg/μL) and PAMAM or PAMAM-FHR (2 mg/mL) at the weight ratio of 4 in a total volume of 20 μL of DMEM medium and further incubated for 24 h. Cell nuclei were stained for 15 min with DAPI. The cells were observed by a confocal microscope (Carl Zeiss, Oberkochen, Germany).

### 2.8. In Vitro Transfection Assay

GBL-14 (1.3 × 10^4^), U373-MG (1.3 × 10^4^), and dermal fibroblasts were cultured in a 96-well tissue plate. Luciferase gene-containing complexes (pJDK-luc and PAMAM derivatives ) were prepared at various weight ratios in a total volume of 20 μL of DMEM medium and then incubated for 30 min at 25 °C. PEI25KD was used as a positive control. Luciferase activity was measured following the manufacturer’s instruction using a luminometer (Lumat LB9507, Oak Ridge, TN, USA). The content of total proteins was analyzed by a bicinchoninic acid (BCA) assay kit (ThermoFisher, MA, USA). 

### 2.9. Expression of Green Fluorescent Protein (GFP)

GBL-14 (5 × 10^3^) and dermal fibroblasts (5 × 10^3^) expressing GFP were seeded in 15 μ-slide 8-well plates for confocal imaging or GBL-14 (2.0 × 10^5^) and dermal fibroblasts (2.0 × 10^5^) were seeded in 6-well tissue culture plates for FACS analysis. Complexes (GFP (1 μg/μL)) and PAMAM derivatives (2 mg/mL) were prepared at the weight ratio of 4 in a total volume of 100 μL of DMEM medium. The samples were incubated for 24 h at 37 °C and analyzed using a confocal microscope. GFP expression was measured with a FACS Calibur system (BD Biosciences, San Jose, CA, USA).

### 2.10. Cell Cycle Analysis

GBL-14 (1.8 × 10^5^) and dermal fibroblasts (1.8 × 10^5^) were cultured in 6-well plates. The cells were treated with pJDK or pJDK-apoptin (1 μg/μL) and PAMAM or PAMAM-FHR (2 mg/mL) at the weight ratio of 4 in a total volume of 100 μL of DMEM medium and then further incubated for 36 h at 37 °C. The cells were then fixed in 70% EtoH for 16 h at 20 °C. Each sample was resuspended in 500 μL phosphate buffer saline and then added to 10 RNase (10 mg/mL) for 30 min at 25 °C. The samples were incubated with propidium iodide (5 mg/mL) in the dark at 25 °C for 10 min prior to measurement by the FACS Calibur system. 

### 2.11. Imaging of Intracellular Trafficking

GBL-14 (5 × 10^3^) and dermal fibroblasts (5 × 10^3^) were seeded into 15 μ-slide 8 wells plates. The cells were incubated with pJDK or pJDK-apoptin (1 μg/μL)) and Alexa Fluor 488-labeled PAMAM and PAMAM-FHR (2 mg/mL) at the weight ratio of 4 in a total volume of 100 μL of DMEM medium for 24 h at 37 °C. The lysosomal compartments were treated with LysoTracker (Invitrogen) for 15 min at 37 °C. The cell nuclei were stained with DAPI for another 15 min at 37 °C. The fluorescence images were acquired using a confocal laser scanning microscope.

### 2.12. Mitochondrial Membrane Potential (MMP) Assay

MMP was determined using a tetramethylrhodamine ethyl ester (TMRE) assay kit (abcam). GBL-14 (1.8 × 10^5^) and dermal fibroblasts (1.8 × 10^5^) were cultured in 6-well tissue culture plates. Complex formation was obtained under the same conditions described for cell cycle analysis. Each sample was exposed to 100 nM TMRE for 15 min at 37 °C and immediately analyzed using FACS.

### 2.13. Annexin V Staining

Apoptosis was determined by a fluorescein isothiocyanate (FITC) annexin V apoptosis detection kit (BD Biosciences, San Jose, USA). GBL-14 (1.8 × 10^5^) and dermal fibroblasts (1.8 × 10^5^) were grown in 6-well tissue culture plates. Complex formation was obtained as described for cell cycle analysis. Annexin V staining was performed according to the manufacturer’s instruction. 

### 2.14. RNA Extract and Real-Time-PCR Quantification

GBL-14 (1.8 × 10^5^) and dermal fibroblasts (1.8 × 10^5^) were grown in 6-well tissue culture plates. Total RNA was isolated by TRIzol reagent following an instruction manual (Invitrogen). First strand cDNA synthesis, real time PCR, and the primers were performed as previously described [28]. 

### 2.15. Measurement of Dynamic Light Scattering (DLS) and Zeta Potential

The complexes (PAMAM (1 mg/mL) or PAMAM-FHR/pJDK or pJDK-apoptin (0.5 µg/μL)) were prepared at the weight ratio of 4 in a total volume of 10 µL of distilled water and incubated for 30 min at 25 °C. The samples were analyzed by DLS (ELS-Z, Osaka, Japan) to obtain the size distribution and zeta potential (Zetasizer Nano, Malvern, Worcestershire, UK) to obtain the surface charge. 

### 2.16. Statistical Analysis

The obtained results are shown as mean ± SD. Statistical analysis was done using *GraphPad prism 5*.

## 3. Results and Discussion

### 3.1. Characteristics of PAMAM-FHR and Complexes with Plasmid DNA

**Scheme 1:**
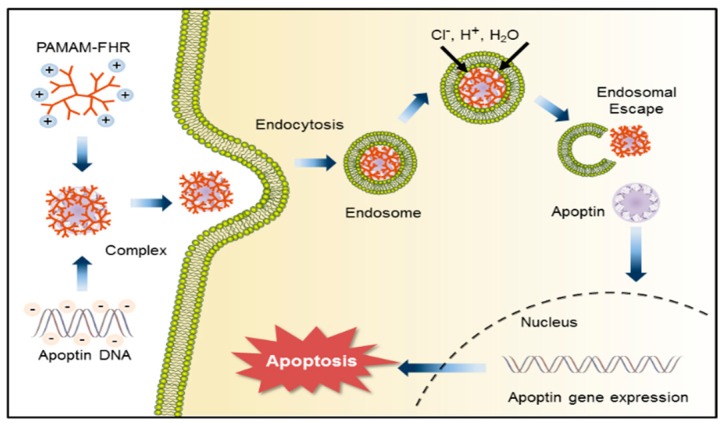
Diagram of complex formation between PAMAM-FHR and apoptin and delivery into the nucleus via endocytosis. Apoptin induces apoptosis in the GBL-14 cell line.

Here, we demonstrated the feasibility of PAMAM-FHR as an effective apoptin gene delivery system in the human primary GBL-14 cell line. The PAMAM-FHR/pJDK-apoptin complex was internalized by cells via endocytosis, after which the apoptin gene was transferred into the nucleus and its expression induced apoptosis. This process is represented in Scheme 1. 

A schematic representation of PAMAM-FHR synthesis is shown in the Supplementary Material, Figure S1. The synthesis of the PAMAM-FHR was confirmed by ^1^H NMR spectroscopy, see Supplementary Material, Figure S2. The yield of the PAMAM-FHR conjugate, calculated by ^1^H NMR spectra, is shown in the Supplementary Material, Table S1.

### 3.2. Characterization of Complexes

**Figure 1:**
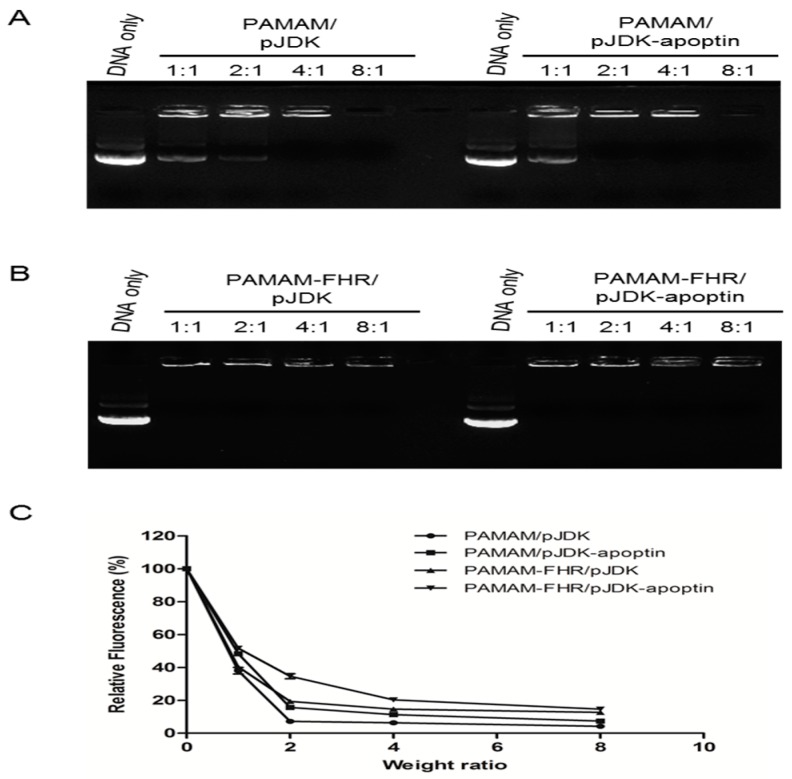
Gel electrophoresis and PicoGreen assays of pJDK-apoptin complexes. (**A**,**B**) each polymer/DNA complex at different weight ratios (0, 1, 2, 4, and 8). Each lane is the weight ratio of polymer/DNA. The DNA concentration was 0.5 µg/µL. (**C**) Complexes were prepared under the same conditions as those used for the gel retardation. The intensity was normalized to 100% in pJDK and pJDK-apoptin plasmid DNA. Results are shown as the mean ± standard deviation, *n* = 3.

Complexation with plasmid DNA is the first process in the nonviral gene delivery process [16]. The formation of the PAMAM-FHR/DNA complexes was examined by agarose gel electrophoresis. At the weight ratio of 2, plasmid DNA (pJDK or pJDK-apoptin) caused a clear inhibition of PAMAM and PAMAM-FHR complex migration, see Figure 1A,B. To quantify these results, a PicoGreen assay was conducted. As shown in Figure 1C, the inhibition of fluorescence resulted from increased weight ratios. Compact DNA complexes were obtained at the weight ratio of 2 for PAMAM and PAMAM-FHR complexes with pJDK or pJDK-apoptin. Notably, because of the additional positive charges, the PAMAM-FHR showed higher condensation than PAMAM, see Supplementary Material, Table S2.

Moreover, to physically characterize the complexes, their size and zeta potential values were analyzed, see Table 1. These results suggested that PAMAM-FHR can function as a polymeric carrier by effectively forming complexes with plasmid DNA in vitro. 

Determined using dynamic light scattering (DLS) measurements at room temperature. Measurements were repeated three times.

### 3.3. Cytotoxicity Assay

**Figure 2:**
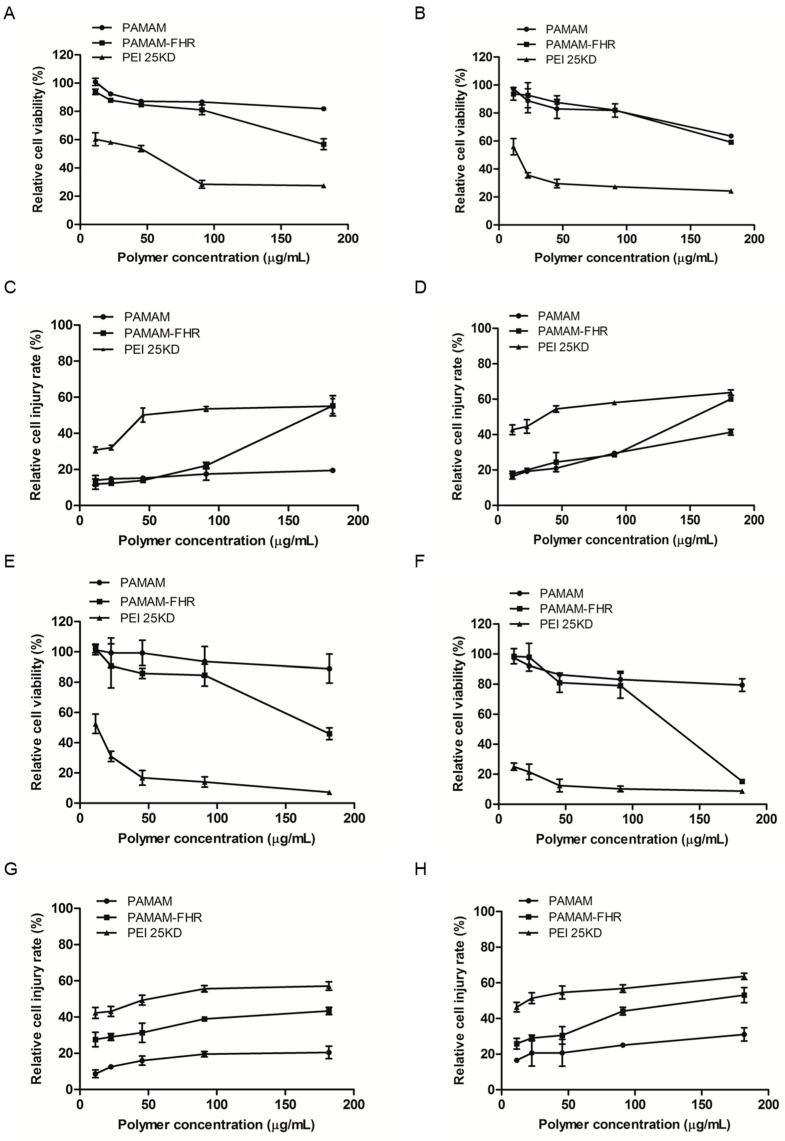

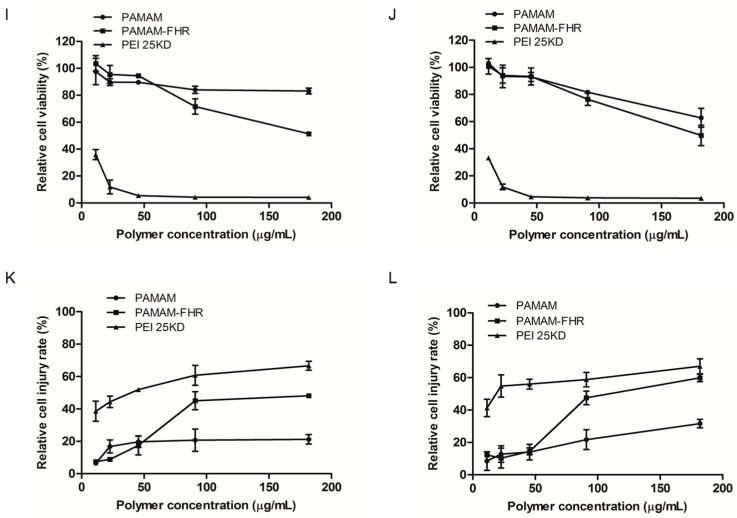
In vitro cytotoxicity assay in PAMAM-FHR-treated cells. (**A**,**B**) GBL-14, (**E**,**F**) U373-MG, and (**I**,**J**) dermal fibroblasts were incubated with different concentrations of the polymers for 24 h (**A**,**E**,**I**) and 48 h (**B**,**F**,**J**). Cytotoxicity was assessed using a WST-1 assay. Results are shown as the mean ± standard deviation, *n* = 3. (**C**,**D**) GBL-14, (**G**,**H**) U373-MG, and (**K**,**L**) dermal fibroblasts incubated under the same conditions as those used for the WST-1 assay. Cell viability was assessed by the lactate dehydrogenase (LDH) assay. After 24 h (**C**,**G**,**K**) and 48 h (**D**,**H**,**L**). Results are shown as the mean ± standard deviation, *n* = 3.

Most cationic polymers are cytotoxic because they can damage the negatively charged plasma membrane or the membrane of cellular compartments [29,30]. To investigate the cytotoxic activity of PAMAM and PAMAM-FHR, PEI25KD was used as a positive control. GBL-14 glioma cells, U373-MG astrocytoma cells, and dermal fibroblasts were treated with increasing doses of polymers for 24 and 48 h. As shown in Figure 2, PEI25KD exhibited high cytotoxicity in all cell lines. As expected, PAMAM displayed negligible toxicity in the three cell lines. After incubation for 48 h, PAMAM-treated GBL-14 cell line and dermal fibroblasts displayed low viability at the highest concentration of 200 µg/mL, see Figure 2B,J. PAMAM-FHR exhibited lower cytotoxic activity compared to PEI25KD in the three cell lines and, except for the high concentration, treatment with PAMAM-FHR did not affect cell viability even as the dose-and-time of the cationic polymer was increased in all the cell lines. However, PAMAM-FHR seemed to alter cell viability depending upon its concentration and exposure time. To confirm this further, an LDH assay was performed [31]. When the polymer dose and treatment period were increased, PAMAM-FHR induced lower toxicity in U373-MG and dermal fibroblasts compared to PEI25KD, see Figure 2G,H,K,L. These results suggested that the PAMAM-FHR owing to its enhanced transfection efficiency is a potential carrier for gene transfer into the glioma cell line. 

### 3.4. Transfection Efficiency In Vitro

**Figure 3:**
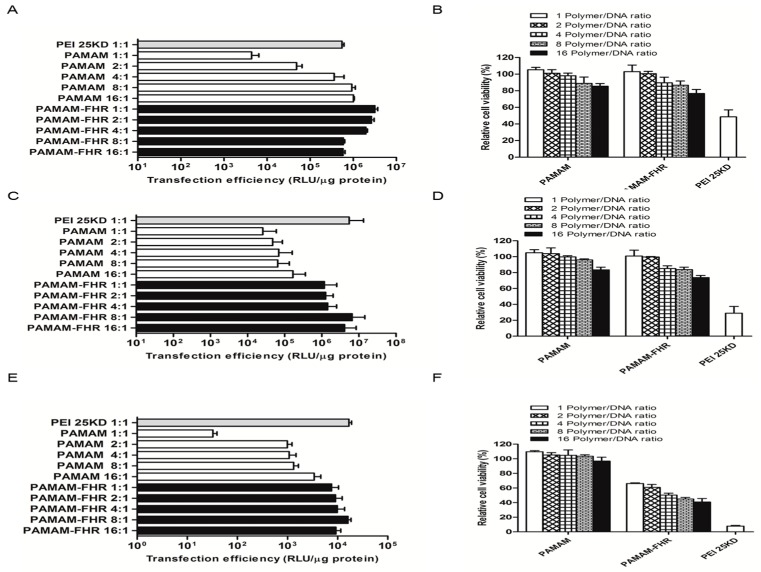
Luciferase activity of PAMAM-FHR. (**A**) GBL-14, (**C**) U373-MG, and (**E**) dermal fibroblasts were treated with each polymer/DNA complex at different weight ratios ranging from 1 to 16. (**B**) GBL-14, (**D**) U373-MG, and (**F**) dermal fibroblasts were incubated with same conditions as those used for luciferase activity assay. The cytotoxicity of complexes was assessed. Results are shown as the mean ± standard deviation, n = 3.

Previous studies have shown that PAMAM-FHR displays a high transfection efficiency and rapid endosomal escape due to its proton sponge effect [18,32]. Therefore, a gene transfection efficiency with this complex was evaluated by a luciferase assay based on a pCN-luc reporter gene system. The cell lines were cultured with the polymers at several weight ratios. As shown in Figure 3A, in the GBL-14 cell line, transfection with PAMAM-FHR was more efficient than with PAMAM up to weight ratio of 4. PAMAM-FHR, hydrophobic amino acid, and phenylalanine have a strong binding with the cell membranes and condense DNA via a hydrophobic chain force [33,34]. Interestingly, the transfection ability of PAMAM-FHR was substantially higher than that of PAMAM in U373-MG and dermal fibroblasts, see Figure 3C,E.

To further test the effect of each polyplex on cell viability, we employed a cell viability assay. As shown in Figure 3B,D, GBL-14 and U373-MG cell lines treated with PAMAM-FHR showed high cell viability independently of the polymer concentration. PAMAM-FHR showed weight ratio-dependent cytotoxic effects compared with that of PAMAM. These results prompted us to further investigate PAMAM-FHR properties. 

**Figure 4:**
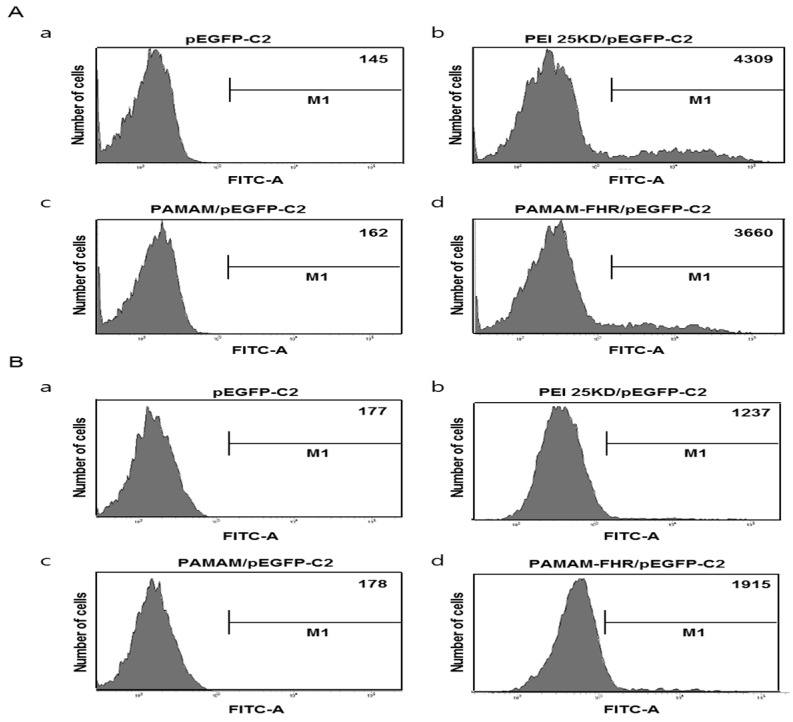
The expression of GFP by the PAMAM-FHR. (**A**) GBL-14 and (**B**) dermal fibroblasts were incubated for 24 h with each polymer/GFP DNA complexes. GFP expression was assessed by FACS analysis.

To confirm the transfection ability of PAMAM-FHR, GFP expression after cell transfection with PAMAM/GFP and PAMAM-FHR/GFP complexes was evaluated. As shown in Figure 4A,B, PAMAM-FHR/GFP resulted in a significantly higher expression compared to PAMAM/GFP. These results confirmed that the PAMAM-FHR is an efficient carrier for gene transfer into the glioma cell line. 

### 3.5. Expression of Apoptin in Cells Treated with PAMAM-FHR/pJDK-Apoptin Complexes

**Figure 5:**
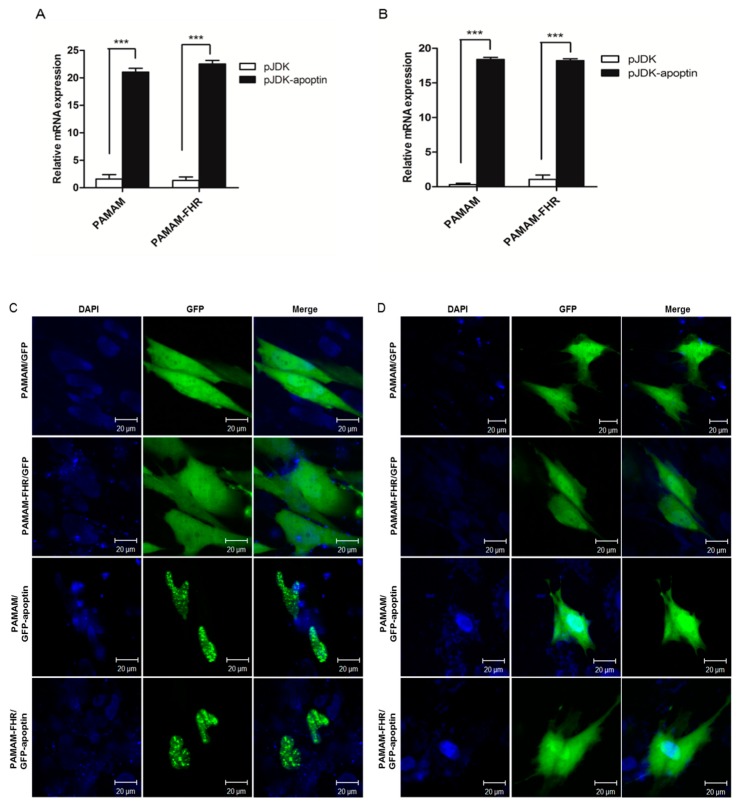
Induction of the apoptin gene expression by PAMAM-FHR. (**A**) GBL-14 and (**B**) dermal fibroblasts were expressed for 24 h with polymer/apoptin complexes at the weight ratio of 4. The expression of the apoptin gene was measured by quantitative Polymerase Chain Reaction (PCR) (q-PCR). Asterisks present statistically significant values. Unpaired *t*-test: *** *p* < 0.001. (**C**) GBL-14 and (**D**) dermal fibroblasts were incubated for 24 h with each polymer/GFP or GFP-apoptin complex and analyzed confocal microscopy.

The transcript levels of apoptin were assessed using q-PCR, see Figure 5A,B. Apoptin expression was highly increased in both cell lines expressed with PAMAM and PAMAM-FHR complexed with the apoptin gene. To examine the subcellular localization of apoptin in cancer and normal cell lines, both GBL-14 and dermal fibroblasts were incubated with PAMAM and PAMAM-FHR complexed with GFP or GFP/apoptin for 24 h. Interestingly, as shown in Figure 5C,D, while GFP-apoptin produced small granules in the nucleus of the GBL-14 cell line. In contrast, it was localized in the cytoplasm of most dermal fibroblasts. 

### 3.6. Intracellular Traffic of PAMAM-FHR/Apoptin Complexes

**Figure 6:**
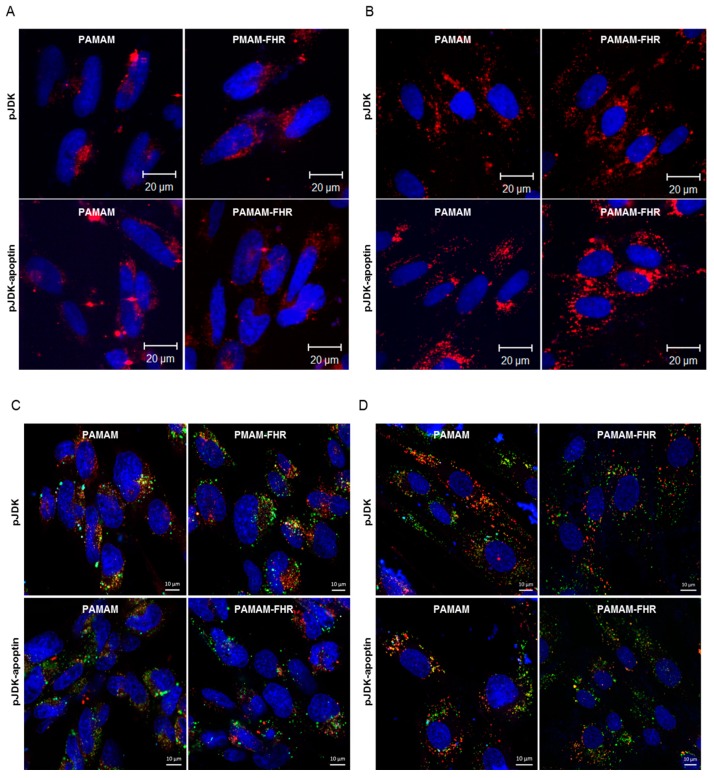
Intracellular localization of PAMAM-FHR. (**A**) GBL-14 and (**B**) dermal fibroblasts were incubated with polymer/Alexa Fluor 546 labeled pJDK or pJDK-apoptin complexes for 24 h. Nuclei were stained before confocal microscopy. (**C**) GBL-14 and (**D**) dermal fibroblasts were incubated with Alexa Fluor 488-labeled each polymer/pJDK or pJDK-apoptin complexes for 24 h. Lysosomal images were incubated with LysoTracker Red for 15 min before confocal microscopy.

The cellular distribution of the PAMAM-FHR/apoptin complexes was further examined by confocal microscopy. As shown in Figure 6A,B, the complexes were mostly cytosolic, but some staining was detectable around the pre-nucleus. Interestingly, PAMAM-FHR produced several red spots inside the nucleus of the GBL-14 cell line. This was likely due to the proton sponge effect provided by phenylalanine, a hydrophobic amino acid, enabling membrane disruption, rapid escape from the endolysosome, and DNA transfer to the nucleus [35,36].

To examine the endo-lysosomal escape ability of the PAMAM-FHR/apoptin, we used LysoTracker Red, which is commonly used to assess the pH in the compartments of living cell lines [37]. As shown in Figure 6C,D, the PAMAM-FHR complex, displaying green fluorescence showed increased cytosolic localization, reflecting rapid escape from the endo-lysosome after intracellular uptake, likely because the histidine residue in the imidazole group conferred a proton buffering capacity. Thus, PAMAM-FHR induced considerably higher gene expression in vitro. 

### 3.7. PAMAM-FHR/pJDK-Apoptin Complexes Induce Apoptosis In Vitro

**Figure 7:**
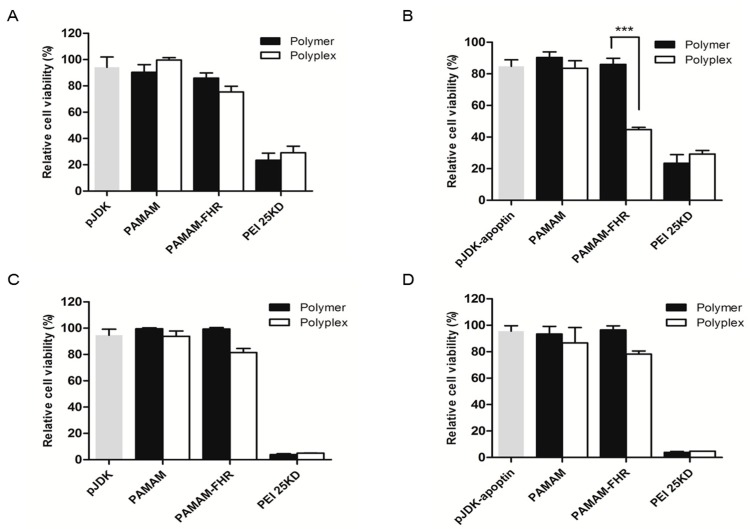
Cytotoxicity induced by PAMAM-FHR/apoptin complexes. (**A**,**B**) GBL-14 and (**C**,**D**) dermal fibroblasts were treated with each polymer/pJDK or pJDK-apoptin for 36 h and cytotoxicity was measured using a WST-1 assay. Results are expressed as mean ± standard deviation (*n* = 3). Asterisks represent statistically significant values. Unpaired *t*-test: ***p < 0.001.

To test the cytotoxic effect of pJDK-apoptin combined with PAMAM or PAMAM-FHR, an EZ-cytotoxicity assay was employed. The viability of the GBL-14 cell line treated with PAMAM-FHR/pJDK-apoptin was significantly reduced compared to that of control cells, treated with PAMAM-FHR/pJDK, see Figure 7A,B. Notably, the viability of dermal fibroblasts was not affected by treatment with PAMAM or PAMAM-FHR combined with pJDK or pJDK-apoptin, see Figure 7C,D. 

**Figure 8:**
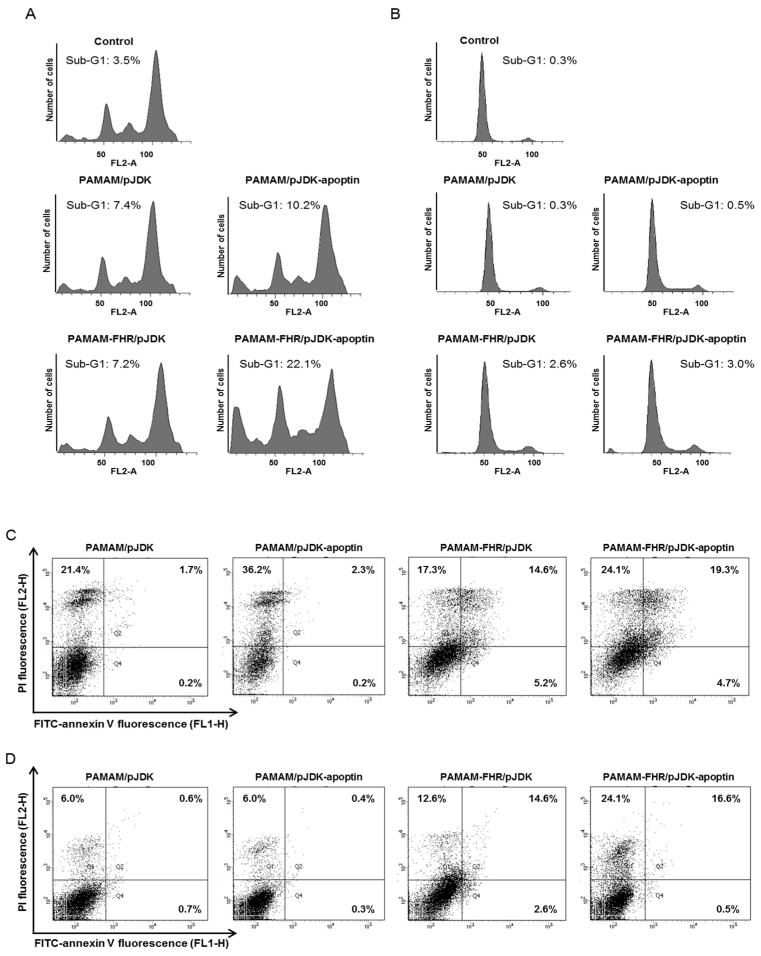
Apoptosis of the PAMAM-FHR/pJDK-apoptin. (**A**,**B**) Cell cycle phase of PAMAM-FHR/pJDK-apoptin using propidium iodide (PI) staining. GBL-14 and dermal fibroblasts were treated with polymer/pJDK or pJDK-apoptin complexes for 36 h prior to flow cytometry. (**C**,**D**) Annexin V staining of PAMAM-FHR/pJDK-apoptin using FACS analysis. Each cell line was incubated under the same conditions as those used for cell cycle analysis. Q3 represents normal cells, Q4: early stage apoptosis, Q2: late stage apoptosis, and Q1: necrosis. PI, propidium iodide.

To verify whether PAMAM-FHR/pJDK-apoptin interfered with the cell cycle, flow cytometry was performed after propidium iodide staining. In the GBL-14 cell line, PAMAM-FHR/pJDK-apoptin complexes were enriched (22.1%) in cells in the apoptotic phase compared to PAMAM/pJDK and PAMAM-FHR/pJDK (7.4% and 7.2%, respectively), as shown in Figure 8A. Conversely, treatment with PAMAM or PAMAM-FHR mixed with pJDK or pJDK-apoptin did not show apoptosis in dermal fibroblasts, see Figure 8B. 

To definitively establish that PAMAM-FHR/pJDK-apoptin induced apoptosis, a FITC-annexin V/PI apoptosis assay was performed. As shown in Figure 8C,D, PAMAM/pJDK-apoptin induced a higher proportion of necrotic cells (36.2%) compared to PAMAM/pJDK (21.4%). Interestingly, compared to PAMAM-FHR/pJDK, PAMAM-FHR/pJDK-apoptin induced late apoptotic cells in the GBL-14 cell line (19.3%). Notably, apoptosis was not observed in dermal fibroblasts after transfection with either PAMAM-FHR/pJDK or PAMAM-FHR/pJDK-apoptin. These results further confirmed that the PAMAM-FHR is an efficient carrier candidate for gene delivery to the GBL-14 cell line and could be employed for gene therapy purposes. 

**Figure 9:**
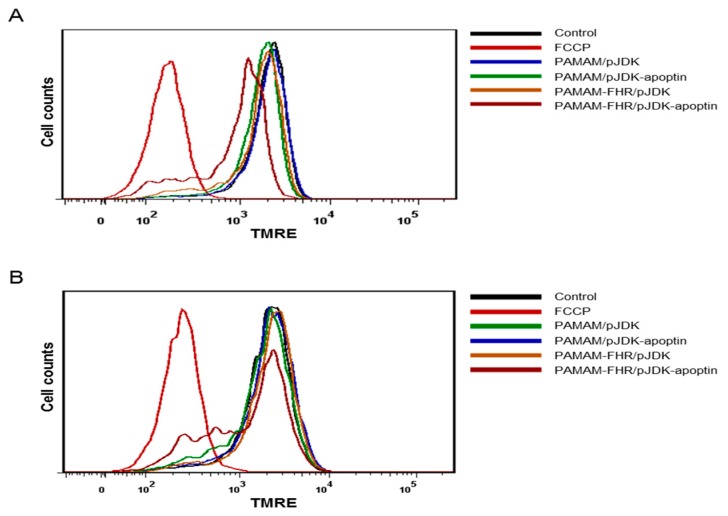
Mitochondrial membrane depolarization by PAMAM-FHR/pJDK-apoptin. (**A**) GBL-14 and (**B**) dermal fibroblasts were incubated with polymer/pJDK or pJDK-apoptin complexes. The cells were analyzed for MMP after TMRE staining and then evaluated by flow cytometry. FCCP was used as a positive control. Results are shown as the mean ± standard deviation, *n* = 3. MMP, mitochondrial membrane potential, FCCP, cyanide 4-(trifluoromethoxy)-phenylhydrazone.

To demonstrate that apoptin expression was associated with intrinsic apoptosis, the mitochondria membrane potential (MMP) was assessed by FACS analysis using TMRE, a lipophilic cationic dye [38]. As shown in Figure 9A,B, FCCP (Carbonyl cyanide 4-(trifluoromethoxy)-phenylhydrazone), an inhibitor of mitochondrial oxidative phosphorylation, was used as a positive control. MMP disruption by FCCP resulted in decreased TMRE fluorescence intensity. Interestingly, PAMAM-FHR/pJDK-apoptin treatment caused a considerable MMP depolarization in the GBL-14 cell line, but not in the dermal fibroblast, whereas PAMAM-FHR/pJDK treatment was ineffective in both cell lines. These results suggested that cell death induced by PAMAM-FHR/pJDK-apoptin was associated with mitochondrial membrane depolarization which, in turn, triggered apoptosis in the glioma cell line. 

## 4. Conclusions

We showed that PAMAM-FHR increased the gene transfection activity compared to PAMAM in a weight-ratio-dependent manner. Cells treated with complexes formed by PAMAM-FHR and plasmid DNA remained viable even with high polymer concentrations. Confocal microscopy indicated that PAMAM-FHR/pJDK-apoptin complexes were more efficiently released from the endolysosome into the cytosol compared to the PAMAM complex and localized around the pre-nucleus. Furthermore, mitochondria-mediated apoptosis was observed in the GBL-14 cell line upon apoptin overexpression. We think that PAMAM-FHR is an effective carrier for gene delivery due to its rapid internalization as well as its ability to induce high gene expression in the GBL-14 cell line. Therefore, the potential of the PAMAM-FHR/pJDK-apoptin complex as a new tool for gene therapy in brain tumors deserves more in-depth investigation. These results form a basis for preclinical applications of PAMAM-FHR in brain tumor therapy. 

## Figures and Tables

**Table 1:** The characteristics of mean diameter, polydispersity, and zeta potential values of polymer/pJDK or pJDK-apoptin complexes.

Complexes	Size (nm)	Polydispersity (PDI)	Zeta Potential (mV)
PAMAM/pJDK	284.6 ± 1.6	0.13	37.8 ± 3.3
PAMAM-FHR/pJDK	185.7 ± 6.3	0.21	40.9 ± 2.7
PAMAM/pJDK-apoptin	239.2 ± 0.7	0.20	36.3 ± 1.3
PAMAM-FHR/pJDK-apoptin	178.7 ± 1.2	0.23	38.5 ± 3.5

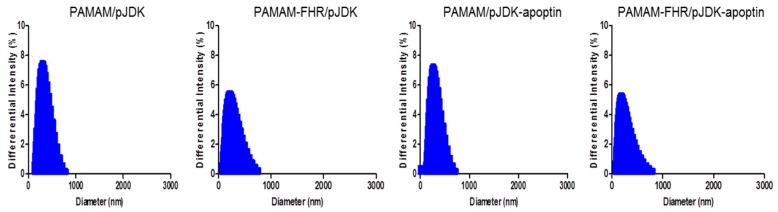

## Supplementary Materials

The following are available online at https://www.mdpi.com/2073-4360/11/2/296/s1. Figure S1. Schematic diagram of synthesis of PAMAM-FHR; Figure S2. 1H-nuclear magnetic resonance (NMR) spectroscopy of the PAMAM (A) and PAMAM-FHR (B). Table S1. Synthesis result of PAMAM-FHR was analyzed by 1H NMR result; Table S2. Number of positive charges present per polymer and per 1.0 μg of polymer.
