# Supplementary material for: Polyplexes of Functional PAMAM Dendrimer/Apoptin Gene Induce Apoptosis of Human Primary Glioma Cells In Vitro
Source: Polymers (Basel). 2019 Feb 10;11(2):296. doi: 10.3390/polym11020296 (PMC6419211; doi:10.3390/polym11020296)
Supplement: Supplementary file 1 [file polymers-11-00296-s001.pdf]

Supporting information

Supplemental Figure 1. Schematic diagram of synthesis of PAMAM-FHR

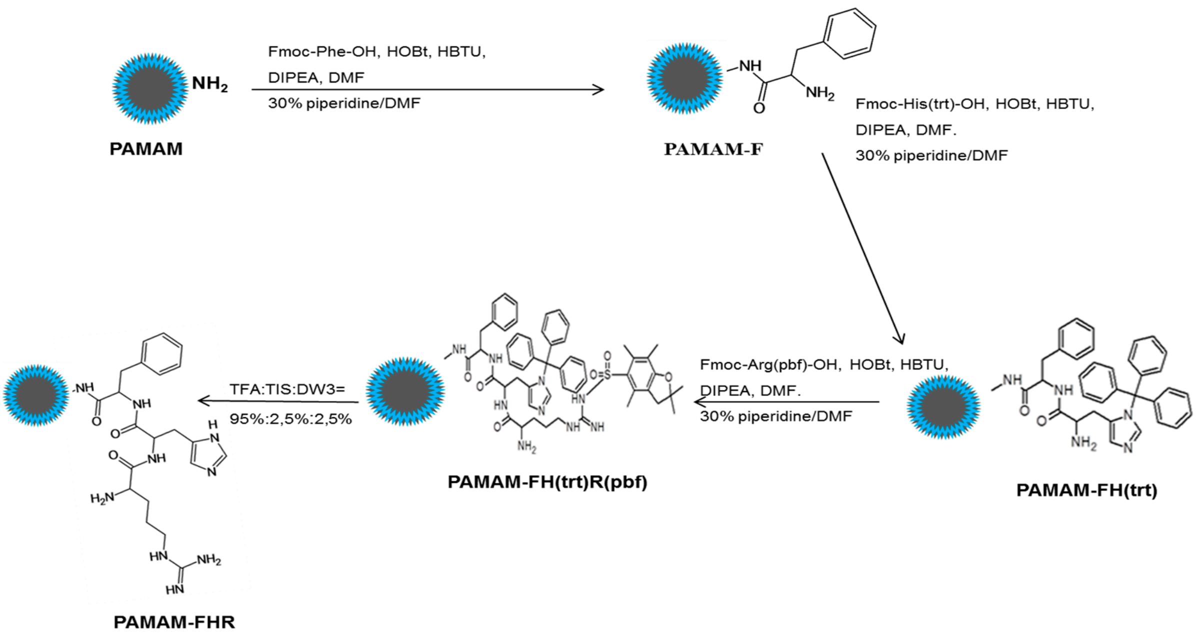

Supplemental Figure 2. **^{1}H-nuclear magnetic resonance (NMR) spectroscopy of the PAMAM** (A) and PAMAM-FHR (B).

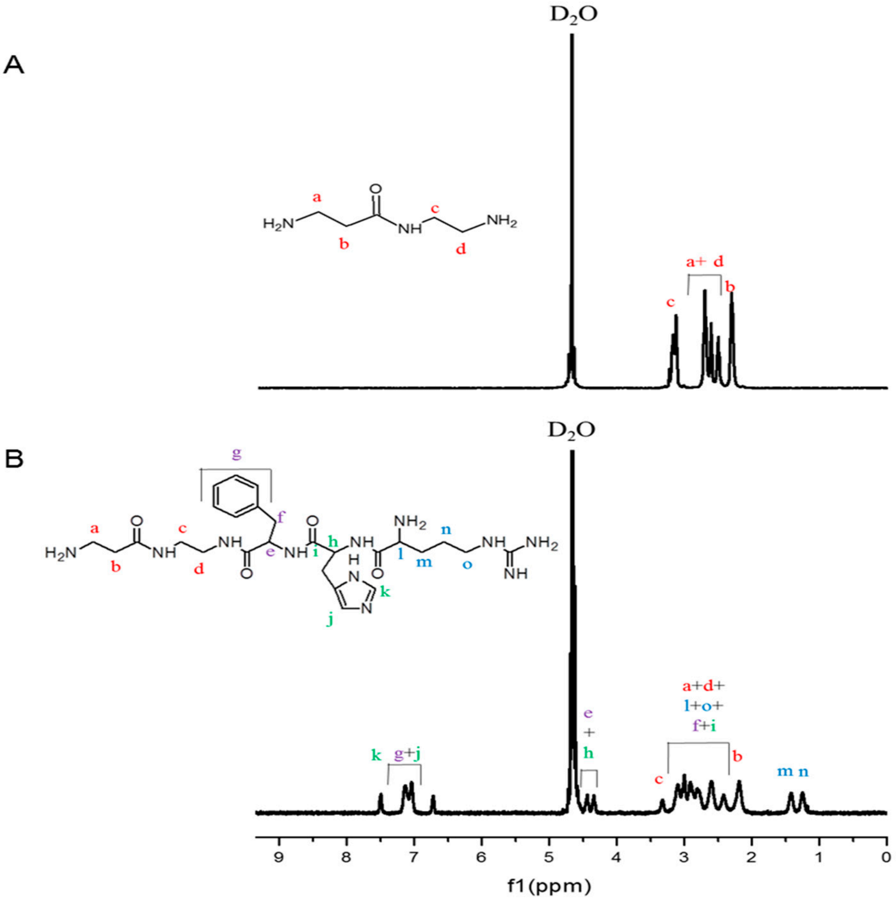

PAMAM δ 2.29 (-NHCH$_2$C**H$_2$**CONHCH$_2$CH$_2$NH- of PAMAM G4 unit), 2.49 (-NHC**H$_2$**CH$_2$CONHCH$_2$CH$_2$NH- of PAMAM G4 unit), 2.6 and 2.69 (-NHCH$_2$CH$_2$CONHCH$_2$C**H$_2$**NH- of PAMAM G4 unit and NC**H$_2$**C**H$_2$**N of PAMAM G4 unit), 3.21 (-NHCH$_2$CH$_2$CONHC**H$_2$**CH$_2$NH- of PAMAM G4 unit) .

PAMAM-FHR δ 2.29 (-NHCH$_2$C**H$_2$**CONHCH$_2$CH$_2$NH- of PAMAM G4 unit), 2.49 (-NHC**H$_2$**CH$_2$CONHCH$_2$CH$_2$NH- of PAMAM G4 unit), 2.6 and 2.69 (-NHCH$_2$CH$_2$CONHCH$_2$C**H$_2$**NH- of PAMAM G4 unit and NC**H$_2$**C**H$_2$**N of PAMAM G4 unit), 3.21 (-NHCH$_2$CH$_2$CONHC**H$_2$**CH$_2$NH- of PAMAM G4 unit), 4.4 (-COC**H**(CH$_2$C$_6$H$_5$)NH- of phenylalanine unit), 2.926 (-COCH(C**H$_2$**C$_6$H$_5$)NH- of phenylalanine unit), 7.2 (-COCH(CH$_2$C$_6$**H$_5$**)NH- of phenylalanine unit), 4.4 (-COC**H**(CH$_2$C$_3$N$_2$H$_3$)NH- of histidine unit), 2.926 (-COCH(C**H$_2$**C$_3$N$_2$H$_3$)NH- of histidine unit),

7.191 (-$COCH(CH_2C_3N_2\mathbf{H_3})NH$- of histidine unit), 7.78 (-$COCH(CH_2C_3N_2\mathbf{H_3})NH$- of histidine unit), 3.265 (-$COC\mathbf{H}(NH_2)CH_2CH_2CH_2NHC(NH)NH_2$ of arginine unit), 1.714 (-$COCH(NH_2)C\mathbf{H_2}CH_2CH_2NHC(NH)NH_2$ of arginine unit), 1.449 (-$COCH(NH_2)CH_2C\mathbf{H_2}CH_2NHC(NH)NH_2$ of arginine unit), 2.6 (-$COCH(NH_2)CH_2CH_2C\mathbf{H_2}NHC(NH)NH_2$ of arginine unit).

Supplemental Table S1. Synthesis result of PAMAM-FHR was analyzed by ^{1}H NMR result.

	Phenylalanine conjugation yield (%)	Histidine conjugation yield (%)	Arginine conjugation yield (%)
PAMAM-FHR	94	99	99

Supplemental Table S2. Number of positive charges present per polymer and per 1.0 μg of polymer.

	**PEI25KD**	**PAMAM**	**PAMAM-FHR**
MW (Da)	25000	14242.22	41665.54
No. of (+)/polymer	581	64	122
No. of (+)/1 μg	1.40×10^{16}	2.71×10^{15}	1.76×10^{15}
